# The Beneficial Effect of Praeruptorin C on Osteoporotic Bone in Ovariectomized Mice via Suppression of Osteoclast Formation and Bone Resorption

**DOI:** 10.3389/fphar.2017.00627

**Published:** 2017-09-12

**Authors:** Xuqiang Liu, Jie-Fen Chin, Xinhua Qu, Haidi Bi, Yuan Liu, Ziqiang Yu, Zanjing Zhai, An Qin, Bin Zhang, Min Dai

**Affiliations:** ^1^Department of Orthopedics, Artificial Joints Engineering and Technology Research Center of Jiangxi Province, The First Affiliated Hospital of Nanchang University Nanchang, China; ^2^Department of Orthopedics, Erasmus University Medical Center Rotterdam, Netherlands; ^3^Shanghai Key Laboratory of Orthopedic Implant, Department of Orthopedics, Shanghai Ninth People’s Hospital, Shanghai Jiao Tong University School of Medicine Shanghai, China

**Keywords:** praeruptorin C, osteoclast, ovariectomized mice, osteoporosis, NF-κB, c-Jun N-terminal kinase

## Abstract

Being a highly prevalent disease, osteoporosis causes metabolism defects. Low bone density, compromised bone strength, and an increased danger of fragility fracture are its main characteristics. Natural compounds have been considered as potential alternative therapeutic agents for treating osteoporosis. In this study, we demonstrated that a natural compound, praeruptorin C (Pra-C), derived from the dried roots of *Peucedanum praeruptorum*, has beneficial effects in suppressing osteoclast formation and resorption function via attenuating the activation of nuclear factor kappa B as well as c-Jun N-terminal kinase/mitogen-activated protein kinase signaling pathways. Moreover, Pra-C was tested in the ovariectomized (OVX) mice, a well-established model of post-menopausal bone loss, and the results indicated Pra-C exerted beneficial effects on inhibiting excessive osteoclast activity and increasing bone mass of OVX mice. Therefore, the protective effects of Pra-C on OVX mice bone are related to its inhibition of osteoclast formation and bone resorption, suggesting that Pra-C is a good potential candidate for osteoporosis treatment.

## Introduction

Bone is a tough and dynamic tissue. It can constantly change its mass as well as its shape to provide the physiological strength needed for an organism’s structural framework. Osteoblasts, osteocytes, and osteoclasts activate to maintain bone homeostasis in a restructuring process called “bone remodeling” (Seeman and Delmas, 2006). Imbalances in the bone remodeling process lead to skeletal diseases. Excessive osteoclastic activity contributes to pathological bone resorption that accounts for most adult skeletal diseases (Rodan and Martin, 2000). Osteoporosis is an example of excessive osteoclast activity (Kong et al., 1999, 2000; Holt et al., 2007), causing low bone mass, compromised bone strength, and increased risk of fragility fracture. Considering this fact, osteoclasts are a key target during the treatment of osteoclast-related osteolytic diseases (Phan et al., 2004). Agents that modulate aberrant osteoclast formation and activity have the potential for use as bone-protective therapies (Zhu et al., 2016).

The monocyte/macrophage lineage is the main source of osteoclasts (Udagawa et al., 1990). Two hematopoietic factors, macrophage colony-stimulating factor (M-CSF) and the receptor activator of nuclear factor kappa B ligand (RANKL), are necessary for osteoclast proliferation and variation, respectively (Lacey et al., 1998; Yao et al., 2002). The binding of RANKL on its receptor, RANK, activates signaling pathways that ultimately lead to osteoclastogenesis. The negative controller of this pathway is osteoprotegerin, the soluble “decoy receptor” for RANKL (Simonet et al., 1997; Lacey et al., 1998; Khosla, 2001). There are at least four distinct signaling pathways activated by RANKL that lead to osteoclastogenesis: nuclear factor kappa B (NF-κB), c-Jun N-terminal kinase (JNK), p38, and extracellular signal-regulated kinase (ERK) (Grigoriadis et al., 1994; Franzoso et al., 1997; Wong et al., 1999; Matsumoto et al., 2000; Hotokezaka et al., 2002; Boyle et al., 2003). On the basis of relevant knowledge and existing outcomes, previous research has created and selected new compounds that are able to inhibit osteoclast formation and activation (Qin et al., 2012; Liu et al., 2014; Qu et al., 2014).

Praeruptorin C (Pra-C) is derived from the dried roots of *Peucedanum praeruptorum* (Peucedani Radix), a therapeutic herb that is frequently utilized in traditional Chinese medicine for treating upper respiratory infections and as an antipyretic, antitussive, and mucolytic agent (Sarkhail et al., 2013). Specifically, it has been shown that Pra-C plays an important role in vascular smooth muscle relaxation (Zhao et al., 1999; Rao et al., 2001), exhibits anti-inflammatory activity in lipopolysaccharide-activated RAW264.7 murine macrophage cells by inhibiting NF-κB and signal transducer and activator of transcription 3 (Yu et al., 2012), and has neuroprotective effects by inhibiting neuronal apoptosis through down-regulation of GluN2B-containing *N*-methyl-D-aspartate receptors (Yang et al., 2013). Given its anti-inflammatory effect via inhibiting NF-κB, and the importance of the NF-κB pathway in osteoclastogenesis (Franzoso et al., 1997; Yu et al., 2012), we hypothesized that Pra-C will provide therapeutic benefits for treating osteoclast-related diseases. The two main aims of this research were to investigate the underlying beneficial effects of Pra-C therapy in a mouse model of osteoporosis and to ascertain the impact of Pra-C on osteoclastogenesis.

## Materials and Methods

### Reagents

RAW264.7 cells were purchased from the American Type Culture Collection (ATCC, Rockville, MD, United States). The alpha modification of minimum essential medium (α-MEM), fetal bovine serum (FBS), and penicillin were acquired from Gibco-BRL (Sydney, Australia). R&D Systems (Minneapolis, MN, United States) provided recombinant soluble human M-CSF and mouse RANKL. In addition, we acquired tartrate-resistant acid phosphatase (TRAP)-staining kit and Pra-C from Sigma-Aldrich (St. Louis, MO, United States) and Meilun Corporation (Dalian, China), respectively; to meet standard requirements, it was essential that the Pra-C was over 98% pure. We purchased antibodies against glyceraldehyde 3-phosphate dehydrogenase (GAPDH), phospho-IκBα, IκBα, ERK, phospho-ERK, JNK, phospho-JNK, p38, and phospho-p38 from Cell Signaling Technology (Cambridge, MA, United States).

### Cell Culture

As previously described (Li et al., 2013), bone marrow monocyte/macrophage (BMM) cells were isolated from the femurs and tibias of 4- to 6-week-old C57BL/6 mice and were cultured in α-MEM containing 30 ng/mL M-CSF, 10% heat-inactivated FBS, and 1% penicillin/streptomycin for 24 h. Then, we separated the adherent cells from the non-adherent cells. The adherent cells were cultivated in a 37°C, 5% CO_2_ incubator until they were confluent. This process usually required 3–4 days. RAW264.7 cells were cultivated in α-MEM with 1% penicillin/streptomycin and 10% FBS at 37°C in a 5% CO_2_ incubator. To prepare BMMs and RAW264.7 cells for experiments, the cells (approximately 90% confluent) were washed three times with phosphate-buffered saline (PBS), and were then trypsinized for approximately 3 min.

### *In Vitro* Osteoclastogenesis Assay

We seeded BMM cells in a 96-well plate at a density of 8 × 10^3^ cells/well in complete α-MEM with 50 ng/mL RANKL and 30 ng/mL M-CSF added. The cells were treated with Pra-C at different concentrations (0, 5, 10, or 20 μM). Every 2 days, the medium was changed, which continued until the osteoclasts matured in the control group. Next, the cells were fixed with 4% paraformaldehyde in PBS twice for approximately 20 min each. Then, a Diagnostic Acid Phosphatase staining kit was used to stain the cells for TRAP. Photographs of the TRAP+ multinucleated cells were taken. The percentage of TRAP+ cell area in each well was measured and the number of TRAP+ cells was determined. TRAP+ cells whose nuclei number was greater than 3 were classified as osteoclasts.

### Cell Viability Assay

To assess Pra-C’s cytotoxic effects, we used cell counting kit-8 (CCK-8, Dojindo Molecular Technologies, Inc., Kumamoto, Japan), following the manufacturer’s protocol. *In vitro*, mature osteoclasts are created by differentiating monocytes/macrophages with M-CSF and RANKL. Using the monocyte/macrophage, osteoclast precursors, we conducted an experiment to assess cell viability with Pra-C treatment. Briefly, BMMs were then added to 96-well plates at 8 × 10^3^ cells/well and cultured for 24 h in complete α-MEM with 30 ng/mL M-CSF. Cells were then treated with varying concentrations of Pra-C (0, 5, 10, 20, 40, 80, 160, or 320 μM) for 2 days. Afterward, 10 μL of CCK-8 substrate was added to every well. The cells were cultured in a 37°C, 5% CO_2_ incubator for another 2 h. The absorbance at 450 nm (650 nm reference) of each well was measured with an ELX800 microplate reader (Bio-Tek, United States). The following formula was used to calculate the cell viability relative to that of the control cells: (experimental group OD - blank OD)/(control group OD - blank OD).

### Cell Apoptosis Assay

We seeded BMM cells in a six-well plate at a density of 2 × 10^5^ cells/well. Different concentrations (0, 5, 10, or 20 μM) of Pra-C were used to treat the cells for 48 h. After washing the cells with cold PBS and collecting the cells by centrifugation, the cell pellets were suspended in 1× annexin-binding buffer. The cell death ratios were evaluated by Annexin V-APC and propidium iodide staining (Becton-Dickinson Company, United States).

### Purification of RNA and RT-qPCR

We used quantitative reverse transcription polymerase chain reaction (RT-qPCR) to analyze gene expression during the formation of osteoclasts. BMMs were seeded in a six-well plate at a density of 1 × 10^5^ cells/well in complete α-MEM with 50 ng/mL RANKL and 30 ng/mL M-CSF. We then treated the cells with different concentrations of Pra-C (0, 10, or 20 μM) until mature osteoclasts formed. The RNeasy Mini kit (Qiagen, Valencia, CA, United States) was used to purify total RNA, following manufacturer’s instructions. cDNA was synthesized from 1 μg total RNA using reverse transcriptase (TaKaRa Biotechnology, Otsu, Japan). Real-time PCR was then conducted with the ABI 7500 Sequencing Detection System (Applied Biosystems, Foster City, CA, United States) and the SYBR Premix Ex Taq kit (TaKaRa). The cycling conditions were as follows: 40 cycles of denaturation at 95°C for 5 s and extension at 60°C for 24 s (Tian et al., 2014). All reactions were performed in triplicate. GAPDH served as the normalizing gene. As depicted previously (Liu et al., 2014), the mouse primer sequences for *GAPDH*, *TRAP*, *V-ATPase a3*, *V-ATPase d2*, Cathepsin K (*Ctsk*), *c-fos*, calcitonin receptor (*CTR*), dendritic cell-specific transmembrane protein (*DC-STAMP*), and nuclear factor of activated T-cells 1 (*NFATC1*) are shown below: *GAPDH* forward 5′-ACCCAGAAGACTGTGGATGG-3′ and reverse 5′-CACATTGGGGGTAGGAACAC-3′, *TRAP* forward 5′-CTGGAGTGCACGATGCCAGCGACA-3′ and reverse 5′-TCCGTGCTCGGCGATGGACCAGA-3′, *V-ATPase a3* forward 5′-GCCTCAGGGGAAGGCCAGATCG-3′ and reverse 5′-GGCCACCTCTTCACTCCGGAA-3′, *V-ATPase d2* forward 5′-AAGCCTTTGTTTGACGCTGT-3′ and reverse 5′-TTCGATGCCTCTGTGAGATG-3′, *Ctsk* forward 5′-CTTCCAATACGTGCAGCAGA-3′ and reverse 5′-TCTTCAGGGCTTTCTCGTTC-3′, *c-fos* forward 5′-CCAGTCAAGAGCATCAGCAA-3′ and reverse 5′-AAGTAGTGCAGCCCGGAGTA-3′, *CTR* forward 5′-TGCAGACAACTCTTGGTTGG-3′ and reverse 5′-TCGGTTTCTTCTCCTCTGGA-3′, *DC-STAMP* forward 5′-AAAACCCTTGGGCTGTTCTT-3′and reverse 5′-AATCATGGACGACTCCTTGG-3′, *NFATC1* forward 5′-CCGTTGCTTCCAGAAAATAACA-3′ and reverse 5′-TGTGGGATGTGAACTCGGAA-3′.

### Resorption Pit Assay

We placed bovine bone slices in a 96-well plate and then seeded BMMs at 8 × 10^3^ cells/well on top in complete α-MEM containing 50 ng/mL RANKL and 30 ng/mL M-CSF. Once mature osteoclasts had formed, we treated with varying concentrations of Pra-C (0, 5, 10, or 20 μM) for another 48 h. Then, with the assistance of mechanical agitation and sonication, the cells adherent to the bone slices, were removed. A scanning electron microscope (FEI Quanta 250) was used to visualize the resorption pits. Image J software (National Institutes of Health, Bethesda, MD, United States) was used to calculate the absorbance areas of the bone slices.

### F-Actin Ring Immunofluorescence

In order to observe the F-actin ring (the ruffled membrane of an osteoclast) formation, we cultivated the BMM-derived osteoclasts on bovine bone slices, and then treated them with different Pra-C concentrations (0, 5, 10, or 20 μM) for 48 h. Following that, we fixed the cells with 4% paraformaldehyde for 20 min. The cells were then permeabilized with 0.1% (v/v) Triton X-100 (Sigma-Aldrich, St. Louis, MO, United States) for 5 min, and incubated with Alexa Fluor 647 phalloidin (Invitrogen, San Diego, CA, United States) for 1 h. The cells were washed with PBS three times. The cell nuclei were then stained with Hoechst 3342 dye (1:5000; Invitrogen), washed with PBS, and mounted on microscope slides with ProLong Gold anti-fade mounting medium (Invitrogen). Fluorescent staining of the F-actin ring was observed with a NIKON A1Si spectral detector confocal system with 10× (dry) lenses equipped.

### Western Blot Analysis

Western blot analysis was conducted according to previously published protocols (Liu et al., 2014). RAW264.7 cells were seeded in six-well plates at 5 × 10^5^ cells/well. After reaching confluence, the cells were pre-treated with or without 20 μM Pra-C for 4 h, and then were stimulated with 50 ng/mL RANKL for 0, 5, 10, 20, 30, or 60 min. Afterward, we washed the cells with PBS and then lysed them using radio-immunoprecipitation assay lysis buffer (50 mM Tris–HCl, 5 mM EDTA, 150 mM NaCl, 1% Triton X-100, 1 mM sodium vanadate, 1 mM sodium fluoride, 1% deoxycholate) with freshly added phenylmethylsulfonyl fluoride (Tian Neng Bo Cai Corp, Shanghai, China). We centrifuged the lysates at 12,000 *g* for 15 min, and collected the supernatants. A bicinchoninic acid assay was used to determine the protein concentrations. As previously reported (Tian et al., 2014), we separated 30 mg of each protein lysate by sodium dodecyl sulfate-polyacrylamide gel electrophoresis (10% gels). The proteins were transferred to polyvinylidene difluoride membranes (Millipore, Bedford, MA, United States). For membranes, they were blocked for 1 h using 5% skim milk powder in TBS-Tween (0.05 M Tris–HCl, pH 7.5, 0.15 M NaCl, and 0.2% Tween-20), and then incubated overnight at 4°C with primary antibodies diluted with 1% skim milk powder in TBS-Tween. The next day, they were washed and then incubated with the corresponding second antibody coupled with IRDye 800CW (molecular weight 1162 Da). Immunoreactive bands were visualized with an Odyssey infrared imaging system (LI-COR, NE, United States).

### Ovariectomized Mouse Model

We used an ovariectomized (OVX) mouse model to analyze the protective effect of Pra-C on the resorption of bone *in vivo*. All animal tests were conducted according to the guiding principles of the Animal Care Committee of Nanchang University. In brief, 20 C57BL/6 mice that were healthy and 8 weeks old (*n* = 20; weight, 21.51 ± 0.87 g) were divided into four groups: non-OVX control mice (sham), OVX mice not treated with drugs (vehicle), OVX mice treated with 5 mg/kg Pra-C (low), and OVX mice treated with 10 mg/kg Pra-C (high) groups. All the mice were housed under controlled temperature (22–24°C) and humidity (50–60%), with a 12 h light/dark cycle and water and food *ad libitum*. The ovaries of the mice were removed as previously described (Moriwaki et al., 2014; Wang et al., 2015). Briefly, after anesthesia with 10% chloral hydrate, retroperitoneal incisions were made ventral to the erector spinae muscles just caudal to the last rib. The ovary and associated fat were located and exteriorized by gentle retraction. To remove the ovary, a 4-0 catgut ligature was placed around the cranial portion of the uterus and uterine vessels. The skin incision was closed with one or two non-absorbable sutures. The low and high concentration mouse groups received intraperitoneal injections with either 5 or 10 mg/kg Pra-C, respectively, every 2 days. As a control, the sham and vehicle groups were injected with 0.9% sodium chloride. After 4 weeks, the mice were euthanized. Their tibias were harvested, fixed in 4% paraformaldehyde for 48 h, washed with PBS, and transferred into 70% ethyl alcohol for Micro-CT scanning or histological study.

### Micro CT Scanning

A high definition Micro-CT (μCT80, Scanco Medical, Switzerland) was used to analyze the fixed tibias. We set the scanning protocol at a 10 μm equidistant definition, 70 kV and 70 μA X-ray energy settings, and a voxel size of 10 μm in three-dimensional (3D) form. Following reconstruction, we chose a region of interest (i.e., 200 slices below the aspect 0.1 mm to the growth plates at the proximal tibia) for further trabecular bone analysis. The volume of bone to tissue (BV/TV, %), trabecular number (Tb.N, 1/mm), trabecular separation (Tb.Sp, mm), and trabecular thickness (Tb.Th, mm) were determined to investigate trabecular structure (Peng et al., 2013).

### Histological and Histomorphometric Study

Following Micro-CT scanning, the tibia samples were decalcified in 10% EDTA with continuous shaking for 3 weeks. After that, they were embedded in paraffin. Tissue sections were prepared and stained with hematoxylin for 5 min and eosin for 2 min at 22–24°C. Other sections were subjected to TRAP staining, according to the instructions provided with the kit. The specimens were visualized, and pictures were taken with a high resolution microscope. Additionally, an area 1 mm in height and width, 0.5 mm below the growth plate and excluding the cortical bone, was chosen to quantify and perform statistical analyses using BioQuant software. The volume of bone, tissue, and TRAP+ multinucleated osteoclasts was quantified in all of the samples.

### Data Analysis

Data from cell and animal experiments were presented as the means ± standard deviations (SD). We conducted data analysis with unpaired Student’s *t*-test and one-way ANOVA using SPSS 13.0 software (SPSS Inc., United States). The unpaired Student’s *t*-test was used when comparing experiments consisting of two groups. And If more than two groups are analyzed, one-way ANOVA with *post hoc* multiple comparisons were used to determine if the groups are statistically different. For these, the data were tested for normality and homogeneity of variance in advance. Briefly, variance was proved to be equal by Levene’s test, and then, pairwise comparison was analyzed according to Student–Newman–Keuls test (SNKq). We considered these differences significant at *P* < 0.05.

## Results

### Pra-C Inhibits Osteoclast Formation *In Vitro*

**FIGURE 1:**
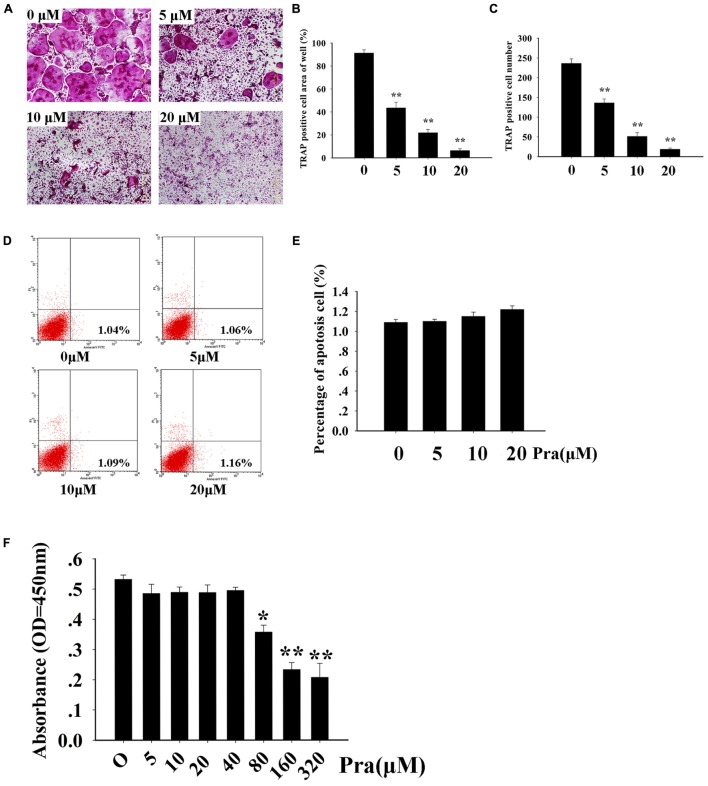
Praeruptorin C (Pra-C) inhibits osteoclast formation without cytotoxic and apoptotic effects. **(A)** Bone marrow-derived macrophages (BMMs) were treated with different concentrations of Pra-C (0, 5, 10, or 20 μM), followed by 30 ng/mL M-CSF and 50 ng/mL RANKL for 5–7 days. Then, cells were fixed with 4% paraformaldehyde and subjected to tartrate-resistant acid phosphatase (TRAP) staining. **(B)** Quantification of the area of TRAP+ cells. **(C)** Quantification of the number of TRAP+ cells. **(D)** BMMs were seeded in six-well plates and treated with different concentrations of Pra-C (0, 5, 10, or 20 μM) for another 48 h, and then subjected to staining with Annexin V-APC and propidium iodide. Cells were analyzed by flow cytometry. **(E)** Quantification of the cell apoptosis rate by flow cytometry. **(F)** BMMs were cultured in a 96-well plate and treated with 30 ng/mL M-CSF, and different concentrations of Pra-C, for 48 h. Cell viability was measured by CCK-8 assay. All plotted data are the means ± SD (*n* = 3). ^∗^*P* < 0.05; ^∗∗^*P* < 0.01, versus 0 μM Pra-C.

First, we studied whether Pra-C has an influence on osteoclast formation *in vitro*. To test this, BMMs were cultured with M-CSF (30 ng/mL) and RANKL (50 ng/mL). BMMs were then treated with varying concentrations of Pra-C (0, 5, 10, or 20 μM) until mature osteoclasts formed. As expected, many TRAP+ multinucleated osteoclasts formed in the group treated without Pra-C (**Figure 1A**). Interestingly, the BMMs treated with Pra-C showed a significant, concentration-dependent decrease in mature osteoclast formation (**Figures 1A–C**). The decrease in mature osteoclast formation could have been caused by a cytotoxic effect of Pra-C. To exclude this possibility, we tested the cell apoptosis rate using flow cytometry. As shown in **Figure 1D**, 1.04% of the cells were apoptotic without Pra-C treatment. Treatment with Pra-C at 5, 10, and 20 μM produced an apoptotic rate of 1.06, 1.09, and 1.16%, respectively. Further quantitative analysis indicated that no significant difference in apoptosis rate was observed even at the highest concentration of 20 μM (**Figure 1E**). Furthermore, to evaluate the cytotoxicity of Pra-C, a CCK-8 assay was performed, and no cytotoxic effect was observed up to 40 μM (**Figure 1F**). Thus, Pra-C inhibits osteoclast formation *in vitro* concentration-dependently with no cytotoxic effect on the cells.

### Pra-C Inhibits RANKL-Induced Gene Expression

**FIGURE 2:**
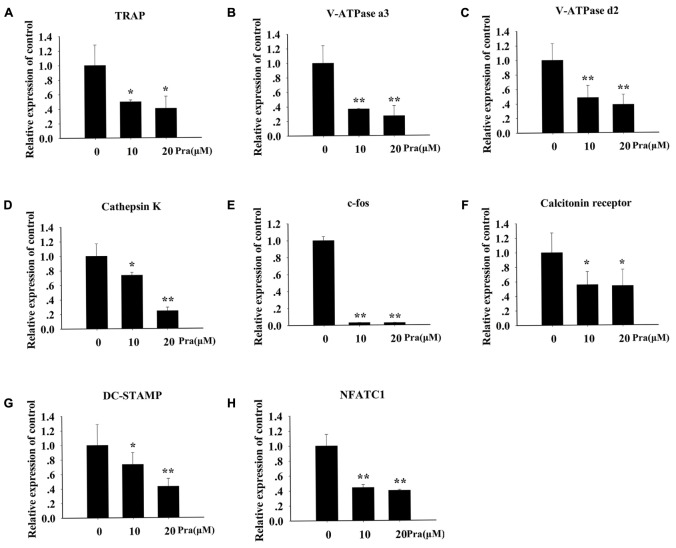
Pra-C suppresses RANKL-induced osteoclast-specific gene expression. BMMs were seeded in six-well plates, and then stimulated with 30 ng/mL M-CSF, 50 ng/mL RANKL, and 10 or 20 μM Pra-C. Once mature osteoclasts were observed, total RNA was extracted and the expression of osteoclast-specific mRNA [**(A)** *TRAP*, **(B)** *V-ATPase a3*, **(C)** *V-ATPase d2*, **(D)** *Cathepsin K*, **(E)** *c-fos*, **(F)** *calcitonin receptor*, **(G)** *DC-STAMP*, and **(H)** *NFATC1*] was measured using quantitative reverse transcription polymerase chain reaction. All experiments were performed at least three times. All plotted data are the means ± SD. ^∗^*P* < 0.05; ^∗∗^*P* < 0.01, versus 0 μM Pra-C.

To explore Pra-C’s influence on the formation of osteoclasts in more detail, the influence of Pra-C on RANKL-induced gene expression was investigated. RANKL-stimulated differentiation of osteoclasts is dependent on numerous signaling pathways, which lead to the upregulation of the expression of particular genes (Boyle et al., 2003). We used RT-qPCR to evaluate RANKL-induced mRNA expression levels of the following osteoclast-linked genes: *TRAP*, *V-ATPase a3*, *V-ATPase d2*, *Ctsk*, *c-fos*, *CTR*, *DC-STAMP*, and *NFATC1*. **Figure 2** shows that the upregulation of all these genes was attenuated by Pra-C treatment. Consistent with previous results, Pra-C also inhibited the upregulation of these genes in a concentration-dependant manner, with the exception of c-fos and CTR. These results confirm the inhibition of osteoclastogenesis by Pra-C by suppressing osteoclast-specific gene expression *in vitro*.

### Pra-C Attenuates Osteoclast Function *In Vitro*

**FIGURE 3:**
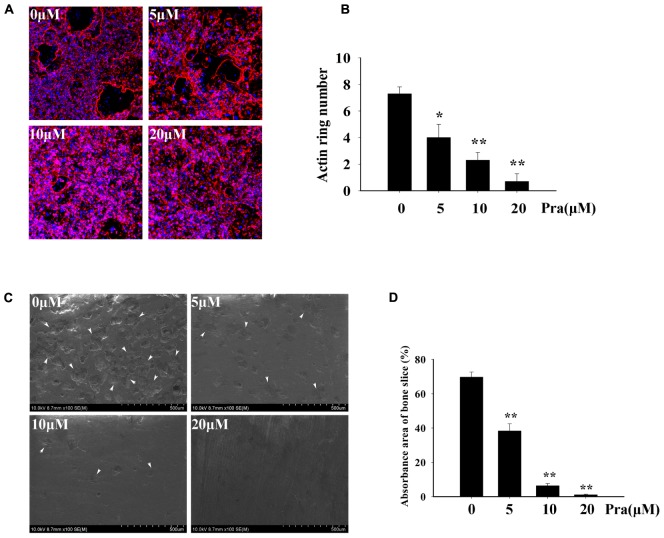
Pra-C impairs F-actin ring formation and inhibits osteoclast bone resorption function. **(A)** BMM-derived osteoclasts were cultured on bovine bone slices and treated with M-CSF (30 ng/mL), RANKL (50 ng/mL), and the indicated concentrations of Pra-C (0, 5, 10, or 20 μM) for another 48 h. Cells were then fixed and stained for F-actin ring formation with Alexa-Fluor 647 phalloidin (red) and imaged on a NIKON confocal system. Representative images are shown. Characteristic F-actin rings were formed in untreated groups. When treated with Pra-C, the formation was suppressed in a concentration-dependent manner. **(B)** Quantification of F-actin ring count using Image J software. **(C)** BMM-derived osteoclasts were cultured on bovine bone slices to conduct a pit resorption assay. Representative SEM images are shown. When subjected to an SEM scanning of bone slices, vast resorption pits were observed in the control group, but they were significantly decreased after treatment with Pra-C. The inhibitory effect occurred in a concentration-dependant manner. The white arrowheads represent resorption pits on bone slices. **(D)** The percentage of absorbance area of bone slices was quantified using Image J software and plotted. All experiments were performed at least three times. All plotted data are the means ± SD. Significant differences were determined using SNKq test. ^∗^*P* < 0.05; ^∗∗^*P* < 0.01, versus 0 μM Pra-C.

For an effective osteoclast function, formation of a highly polarized F-actin ring is indispensable (Wilson et al., 2009). Hence, we explored the influence of Pra-C on F-actin ring formation. For the untreated control group, we observed a typical F-actin ring using Alexa Fluor 647 Phalloidin and confocal microscopy (**Figure 3A**). In the groups treated with varying concentrations of Pra-C (5, 10, or 20 μM), Pra-C severely affected F-actin ring formation and morphology. It was observed that F-actin formation was suppressed in a concentration-dependant manner, similar to that observed with the other assays (**Figures 3A,B**).

Our previous results indicated that Pra-C inhibits the formation of osteoclasts *in vitro*. Therefore, we hypothesized that Pra-C would also attenuate osteoclast function *in vitro*. To test this, we seeded BMMs onto bovine bone slices and stimulated them with M-CSF and RANKL. Once mature osteoclasts formed, they were treated with varying concentrations of Pra-C (0, 5, 10, or 20 μM) for 48 h. The bone slices were then analyzed with a scanning electron microscope. **Figure 3C** shows many bone resorption pits in the control group, while the quantity of bone resorption pits is lower in the Pra-C-treated groups. As shown in **Figure 3D**, there is a large bone resorption area (∼70%) on the bone slice surface in the control group. However, the resorption area significantly reduced to ∼40 and ∼10% with Pra-C concentrations of 5 and 10 μM, respectively. We observed almost no resorption pits when BMMs were treated with a concentration of 20 μM Pra-C. These results show that both osteoclast formation and osteoclast function are inhibited by Pra-C *in vitro*.

### Pra-C Suppresses RANKL-Induced Activation of NF-κB and JNK Signaling Pathways

**FIGURE 4:**
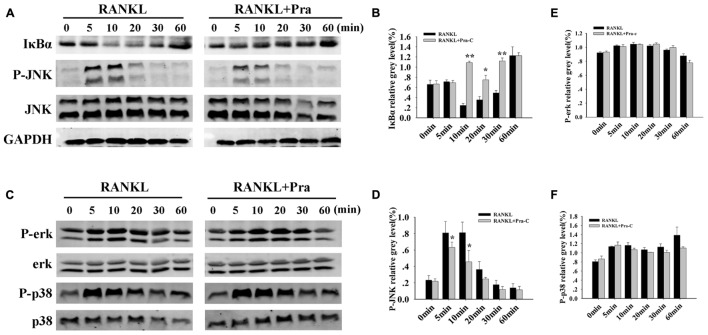
Pra-C impairs RANKL-induced NF-κB and JNK signaling pathways. **(A)** Total protein extracted from RAW264.7 cells that had been cultured with or without 20 μM Pra-C for 4 h, followed by 50 ng/mL RANKL for the indicated times was subjected to western blotting with specific antibodies against IκBα, phospho-JNK, JNK, and GAPDH. In the control group, IκBα rapidly degrades, and JNK is rapidly phosphorylated within 10 min. IκBα degradation and JNK phosphorylation were greatly impaired in the presence of Pra-C. **(B)** The band intensities corresponding to IκBα were quantified and normalized to GAPDH levels using Image J software, and are plotted. **(C)** Western blotting was used to analyze phospho-ERK, ERK, phospho-p38, and p38. No difference was observed in the phosphorylation of these two cascades between the control and Pra-C-treated groups. **(D)** JNK phosphorylation was quantified and normalized to GAPDH levels, and significant differences were shown between the control and Pra-C-treated groups. No significant differences were found when ERK phosphorylation **(E)** and p38 phosphorylation **(F)** were quantified. Values are expressed as means ± SD (^∗^*P* < 0.05; ^∗∗^*P* < 0.01 compared to control group). All the experiments were performed at least three times and representative blots are shown in panels **(A,C)**.

To analyze potential mechanisms of inhibitory influence of Pra-C on the formation of osteoclasts and their functions *in vitro*, RANKL-induced signaling cascades were explored using western blotting. The binding of RANKL to its receptor, RANK, activates NF-κB and MAPKs (Nakashima et al., 2012). Activating the NF-κB pathway is crucial for osteoclast formation. Furthermore, the three major MAPK signaling cascades (p38, ERK, and JNK) have also been shown to be crucial for inducing and activating osteoclast formation and function. As expected after RANKL stimulation, IκBα in the control group is rapidly degraded within minutes. In comparison, IκBα degradation was greatly impaired in the presence of Pra-C (**Figures 4A,B**). This implies a disruption in the NF-κB pathway during osteoclast formation. JNK phosphorylation also seemed to be inhibited, and quantitative analysis indicated a significantly decreased phospho-JNK signal after treatment with Pra-C, compared to that of the control group (**Figures 4A,D**). This suggests that Pra-C also inhibits the JNK signaling pathway during osteoclast formation. The two remaining MAPK cascades (ERK and p38) showed no difference in phosphorylation between the control group and the Pra-C-treated group (**Figures 4C,E,F**). These results indicate that Pra-C attenuates osteoclast formation by inhibiting NF-κB and JNK pathways without influencing ERK and p38 pathways.

### Pra-C Suppresses Bone Loss in OVX Mice

**FIGURE 5:**
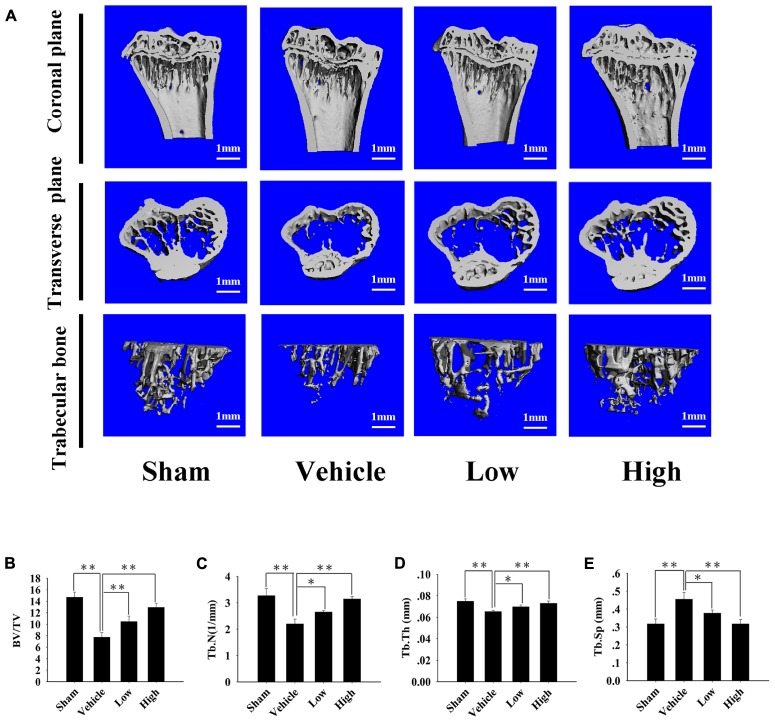
Pra-C prevents ovariectomized (OVX) mouse bone loss. **(A)** Right tibias of all mice were harvested and subjected to a high-resolution Micro-CT scanning. Representative three-dimensional (3D) reconstructed images are shown. Severe bone loss was observed in vehicle-treated OVX mice. Compared to that of the vehicle-treated group, bone mass was increased in both Pra-C-treated groups. **(B–E)** Bone volume to tissue volume (BV/TV, %), trabecular number (Tb.N, 1/mm), trabecular thickness (Tb.Th, mm), and the trabecular separation (Tb.Sp, mm) of each sample were measured and analyzed. All plotted data are the means ± SE (*n* = 5). ^∗^*P* < 0.05; ^∗∗^*P* < 0.01 compared to vehicle group.

After examining the influence of Pra-C on osteoclasts *in vitro*, we investigated the influence of Pra-C on preventing bone loss *in vivo* using an OVX mouse model. In **Figure 5A**, Micro-CT with 3D reconstruction of the tibia shows obvious bone loss in the vehicle group (vehicle-treated OVX mice). There is also more trabecular bone in the group treated with a high concentration of Pra-C (10 mg/kg) compared to that in the group treated with a low concentration of Pra-C (5 mg/kg). Furthermore, **Figures 5B–E** shows the histomorphometric analyses that were performed to determine BV/TV (%), Tb.N (1/mm), Tb.Sp (mm), and Tb.Th (mm). Compared to that of the sham group, all four parameters revealed significantly more bone resorption in vehicle group (*P* < 0.01). Moreover, BV/TV, Tb.N, and Tb.Th were obviously higher in the Pra-C-injected groups than in the vehicle group, while Tb.Sp was remarkably lower in the Pra-C-injected groups than in the vehicle group. Additionally, when comparing to the sham group, BV/TV and Tb.N was still lower in the two Pra-C-treated groups, and a significant difference was observed (*P* < 0.05). Otherwise, referring to Tb.Th and Tb.Sp, no obvious difference was represented between sham and two Pra-C-treated groups (*P* > 0.05). Based on these results, it is concluded that Pra-C can partially prevent OVX-induced bone loss.

**FIGURE 6:**
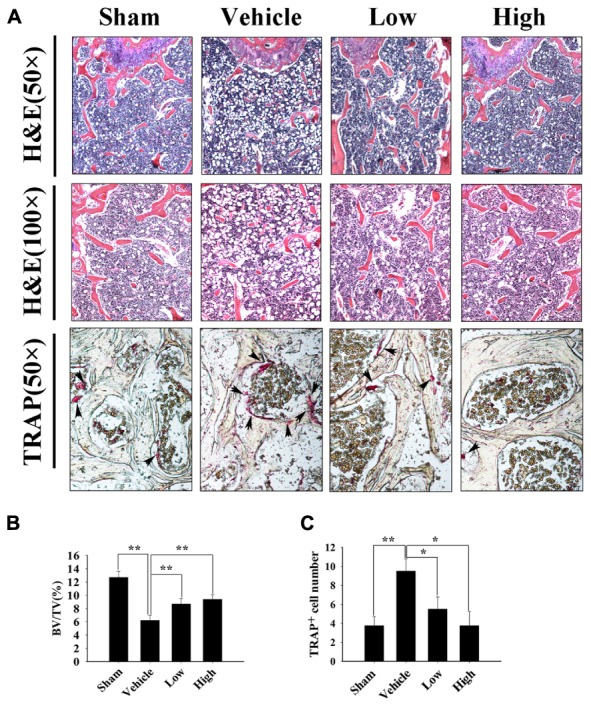
Pra-C prevents ovariectomized (OVX) mouse bone loss as assessed with histological and histomorphometric analyses. **(A)** Decalcified bone tissue was paraffin-embedded and sectioned for hematoxylin and eosin, as well as tartrate-resistant acid phosphatase (TRAP) staining. Representative microscopic images are shown at the indicated magnification. Increased bone loss and TRAP-positive osteoclasts were observed in vehicle-treated OVX mice, whereas Pra-C treatment reversed the pathology, bone loss, and the number of mature osteoclasts was obviously reduced. The black arrowheads represent TRAP-positive osteoclasts observed on the surface of trabecular bone below growth plate. **(B)** Bone volume to tissue volume (BV/TV) and **(C)** the number of TRAP-positive mature osteoclasts was counted in each sample using BioQuant software. All plotted data are the means ± SE (*n* = 5). ^∗^*P* < 0.05; ^∗∗^*P* < 0.01 versus vehicle group.

Further histological and histomorphometric analyses confirmed the protective effect of Pra-C on preventing bone loss. An increased mature osteoclast number was observed in the vehicle-treated OVX group, leading to significant bone loss and impaired trabecular microarchitecture (**Figure 6A**). Compared to that of the OVX group, Pra-C-treated groups showed a preservation of bone mass and decreased mature osteoclast numbers (**Figures 6B,C**). Quantitative analysis of histomorphometric vectors and BV/TV showed similar results as the Micro-CT scanning did (**Figure 6B**).

## Discussion

Bone homeostasis is maintained by two coordinated procedures, osteoblast bone formation and osteoclast bone absorption (Boyle et al., 2003; Teitelbaum and Ross, 2003; Horne et al., 2005). When this balance is disturbed, increases or decreases in bone mass will occur, and bone structure is altered. Osteoclasts, as the only cells capable of degrading bone matrix, play a critical role in this metabolic process. Perturbations, such as osteoporosis, primary and secondary bone tumors, aseptic loosening of arthroplasty, and rheumatoid arthritis, are all caused by over-activation of osteoclasts and the resulting excessive bone destruction (Zaidi, 2007). Because of this, osteoclasts are considered a key target in treating osteoclast-related osteolytic diseases (Phan et al., 2004).

One disease related to osteoclasts, osteoporosis, is becoming a global health concern that causes huge medical, economic, and social burdens. Treatments for osteoporosis currently focus on two different classes of medication, antiresorptive agents and anabolic agents (Amugongo et al., 2014). Approved and recommended antiresorptive agents include bisphosphonates, selective estrogen receptor modulators, and monoclonal antibodies against RANKL (Denosumab). Although bone turnover is decreased and bone microarchitecture is modified using these antiresorptive agents, they are far from ideal because of their side effects. Treatment with selective estrogen receptor modulators may increase the risk of stroke and cardiovascular events (Barrett-Connor et al., 2006), while prolonged use of bisphosphonates has been associated with severe gastrointestinal discomfort, atypical femoral fracture, and osteonecrosis of the jaw (Fleisch, 2003; Yarom et al., 2007; Lenart et al., 2008; Abrahamsen et al., 2009). Denosumab, a humanized monoclonal antibody of RANKL, shows potent inhibitory effects on bone resorption and rapid improvement of bone mineral density. However, osteonecrosis of the jaw has been observed in registration trials (six cases in 4450 patients) (Bone et al., 2013), and its long-term efficacy remains to be confirmed. Teriparatide (recombinant human parathyroid hormone), an anabolic agent for osteoporosis, produces the largest increase in bone mineral density, when compared to that of any other osteoporosis treatment (Neer et al., 2001). However, its high cost and inconvenient daily injections limit its usefulness to only severe osteoporosis patients. Romosozumab, a humanized monoclonal antibody, is associated with bone formation, increased bone mineral density, and suppressed bone resorption, which is different from any other anabolic agent (McClung et al., 2014; Ishibashi et al., 2017). The novel effects of Romosozumab make it a promising agent in treating osteoporosis (Clarke, 2014), but its efficacy and safety still need to be evaluated in a Phase III study. Considering the current therapeutic options, exploring available agents to treat osteoporosis is an urgent clinical and social requirement.

Natural compounds are potential alternative therapeutic agents for treating osteoporosis. Many investigators have focused on these compounds because their various bioactivities can be useful in treating human diseases. Several natural herbs, including icaritin, maslinic acid, and lycorine, show anti-osteoporosis properties (Li et al., 2011; Peng et al., 2013; Chen et al., 2015). In our research, we demonstrated that Pra-C, which is derived from the dried roots of *P. praeruptorum*, showed beneficial effects in preventing bone loss via suppression of osteoclast formation and resorption activity. According to previous findings, praeruptorins (A–D) have been adopted in traditional Chinese medicine to treat cough and upper respiratory infections, because of their antipyretic, antitussive, and mucolytic properties (Xiong et al., 2012; Sarkhail, 2014). Additionally, Pra-C exhibits anti-inflammatory activity and neuroprotective effects due to the suppression of NF-κB signaling and the down-regulation of GluN2B-containing *N*-methyl-D-aspartate receptors (Yu et al., 2012; Yang et al., 2013). Given the suppression of NF-κB cascades and their importance in osteoclast formation, we hypothesized that Pra-C would possess an additional property of inhibiting osteoclast differentiation via this mechanism. Thus, we focused our attention on the potential influence of Pra-C on osteoclast formation, function, and the therapeutic effect toward osteoclast-related diseases.

In this study, we clarified for the first time that Pra-C suppresses osteoclastogenesis concentration-dependently, using concentrations from 5 to 20 μM, without showing cytotoxicity or apoptosis. Furthermore, osteoclast-specific gene expression, including *TRAP*, *V-ATPase a3*, *V-ATPase d2*, *Ctsk*, *c-fos*, *CTR*, *DC-STAMP*, and *NFATC1*, were also down-regulated after treatment with Pra-C. The mature, multinucleated osteoclast cell body is polarized, and it undergoes internal structural changes, such as actin cytoskeleton rearrangements, during differentiation (Boyle et al., 2003). A notable feature of polarized osteoclasts is a ruffled membrane (also called the F-actin ring), which contains numerous vacuolar proton pumps (H^+^-V-ATPase), and is associated with osteoclast bone absorption activity (Boyle et al., 2003; Teitelbaum and Ross, 2003). With the aim of evaluating the effect of Pra-C on osteoclast resorption function, F-actin immunofluorescence and bone resorption assays were conducted. The results indicated that Pra-C impairs F-actin formation and induces a generalized cytoskeletal membrane disruption. Because of the inhibition of osteoclast differentiation and disruption of the F-actin ring, the quantity and area of bone absorption pits were radically reduced with the treated cells. Regarding the mechanism underlying Pra-C’s effect on osteoclast resorptive function, we hypothesize that the down-regulation of NFATC1 expression impairs cytoskeletal membrane integrity and formation of F-actin, resulting from the inhibition of integrin αvβ3 and c-Src tyrosine kinase, both of which are downstream of NFATC1 activation (Miyazaki et al., 2004; Crotti et al., 2006; Nakamura et al., 2007). However, the precise mechanisms of the pathways remain to be revealed in future investigations.

During the procedures of osteoclast differentiation, RANKL binding to its receptor, RANK, on osteoclast precursor cells triggers a rapid activation of several critical signaling cascades, including NF-κB, JNK, ERK, and p38, due to recruitment of the adaptor protein, TRAF6 (Nakashima et al., 2012; Ouyang et al., 2014). As a result of these activated pathways, extracellular stimuli are transmitted from the cell surface to the nucleus. Former investigators have implicated p38-regulated microphthalmia-associated transcription factor (Matsumoto et al., 2000), ERK-induced activation of c-fos (Monje et al., 2005), and JNK phosphorylated down-stream factors, including c-fos and c-Jun, which combine to form the activator protein-1 (AP-1) heterodimer (Grigoriadis et al., 1994; Kobayashi et al., 2001). Additionally, IκB degradation after IκB kinase (IKK) phosphorylation is another important event during osteoclastogenesis, resulting in NF-κB P65/RelA translocation to the nucleus. Consistent with previous reports, our study clearly indicated that the NF-κB pathway was suppressed by Pra-C, and confirmed by the observation that IκB degradation was significantly attenuated. Unexpectedly, the results showed that the RANKL-induced JNK cascade was also impaired after treatment with Pra-C. Therefore, related to the attenuation of NF-κB and JNK cascades, disrupted osteoclast formation was observed during the differentiation procedures when the BMMs were induced with RANKL.

Pra-C’s suppressive effect on osteoclastogenesis and bone resorption *in vitro* suggests that it is possible to use Pra-C as an antiresorptive agent for treating osteolytic disease. Osteoporosis is one of the most prevalent diseases resulting from estrogen deficiency and the resulting over-activation of osteoclasts. Therefore, we further studied the effect of Pra-C *in vivo* using a model of post-menopausal bone loss. Tibias separated from the mice were subjected to Micro-CT scanning, hematoxylin and eosin staining, and osteoclast-specific TRAP staining. Our results suggest that Pra-C is beneficial for preventing bone loss and improving trabecular bone microarchitecture by suppressing osteoclast formation. In conclusion, this research demonstrated that Pra-C has a beneficial effect on the bone of OVX mice, resulting from inhibition of osteoclast formation and bone resorption. Our research also indicated that this inhibitory effect was at least partly due to the inhibition of RANKL-induced NF-κB activation, as well as JNK signaling pathways. Therefore, Pra-C’s anti-resorptive properties make it good potential candidate for osteoporosis treatment. However, further researches are still required before it can be developed as a therapeutic agent.

## Ethics Statement

This study was carried out in accordance with the recommendations of Experimenting on animals, Research Ethics Committee of the Shanghai Ninth People’s Hospital. The protocol was approved by the Research Ethics Committee of the Shanghai Ninth People’s Hospital.

## Author Contributions

MD and BZ designed most of the experiments. XL, J-FC, and XQ were primarily responsible for carrying out all experimental procedures. XL, J-FC, XQ, and HB carried out the cell experiments. XL, J-FC, YL, ZY, and ZZ performed the animal surgery. XL analyzed the data. XL and J-FC wrote the paper. AQ helped design the experiments.

## Conflict of Interest Statement

The authors declare that the research was conducted in the absence of any commercial or financial relationships that could be construed as a potential conflict of interest.
